# Institutional Amenities

**Published:** 1898-11-19

**Authors:** 


### Editor's Letter-Box.

[Our Correspondents are reminded that prolixity is a great bar to publication, and that brevity of style and conciseness of statement greatly facilitate early insertion.]

### INSTITUTIONAL AMENITIES.

"Another Hospital Secretary" writes: I have read with interest the article in your paper on the above. Like your correspondents, I am visited by gentlemen of the press from time to time, and was "interviewed" by one a few weeks ago. I am pleased to state that I usually find them sensible, straightforward men, who show every desire to take up as little time as possible. On my part I endeavour to be courteous and obliging to all, as we, without doubt, should. On reading your correspondent's letter in last week's issue I immediately felt "familiar" with the facts mentioned by him; and now, with the letter from the *Friday Review* before me, have no doubt of the identity of the journal in question. I received a letter in May last, stating exactly as shown in the extracts of your correspondent. I replied, offering every facility for the desired interview. "Hospital Secretary and Pressman" has been favoured with his, but I, after six months, am still waiting for mine! The appearance of the *Friday Review's* series of articles on the London hospitals and charities is awaited with curiosity by your obedient servant.

